# CYP8B1 inhibits hepatocellular carcinoma progression by repressing PAK4 transcription through inhibition of nuclear translocation of u-STAT1

**DOI:** 10.1038/s41419-025-08393-3

**Published:** 2025-12-31

**Authors:** Le Xu, Lin Xiong, Jingping Yuan, Yukai Chen, Fangfang Chen, Siyu Wang, Ximing Xu

**Affiliations:** 1 Cancer Center, Renmin Hospital of Wuhan University, Wuhan, Hubei China; 2 Department of pathology, Renmin Hospital of Wuhan University, Wuhan, Hubei China

**Keywords:** Tumour-suppressor proteins, Nuclear receptors

## Abstract

Primary and secondary bile acid (BA) levels are elevated in patients with hepatocellular carcinoma (HCC). BAs are important signaling molecules that regulate CYP8B1 expression by targeting nuclear and membrane receptors. In this study, we aimed to determine the function of CYP8B1 in HCC. Examination of HCC tissue and bioinformatic analysis revealed that CYP8B1 expression is downregulated in HCC tissues and is associated with good prognosis. Cholic acid promoted Huh7 cell proliferation and migration by inhibiting CYP8B1 expression. Both in vitro and in vivo, CYP8B1 inhibited the proliferation, invasion, and migration of HCC cells. Nanopore long-read RNA-sequencing analysis identified PAK4 as a potential target of CYP8B1, and the MAPK pathway was associated with CYP8B1 expression. CYP8B1 inhibited PAK4 expression and Raf/MEK/ERK phosphorylation. Tissue microarray analysis also verified a strong correlation between CYP8B1 and PAK4 expression. In vitro Cell Counting Kit 8 assays and in vivo orthotopic liver tumor model analyses showed that CYP8B1 restores sorafenib sensitivity in resistant HC, suggesting its potential as a therapeutic target. IP-MS of CYP8B1 and transcription factor prediction of PAK4 revealed STAT1 as a potential transcription factor for PAK4, which may directly bind to CYP8B1. Chromatin immunoprecipitation confirmed that u-STAT1 directly binds to the PAK4 promoter, not p-STAT1. Overall, CYP8B1 binds to u-STAT1 in the cytoplasm, reducing the translocation of u-STAT1 from the cytoplasm to the nucleus, thereby inhibiting the transcription of PAK4 and ultimately inhibiting the phosphorylation of Raf/MEK/ERK. Our findings indicate that the CYP8B1/PAK4 axis is important in HCC progression and elucidate the mechanism by which BAs promote HCC. Thus, CYP8B1 is a potential therapeutic target for the clinical treatment of HCC.

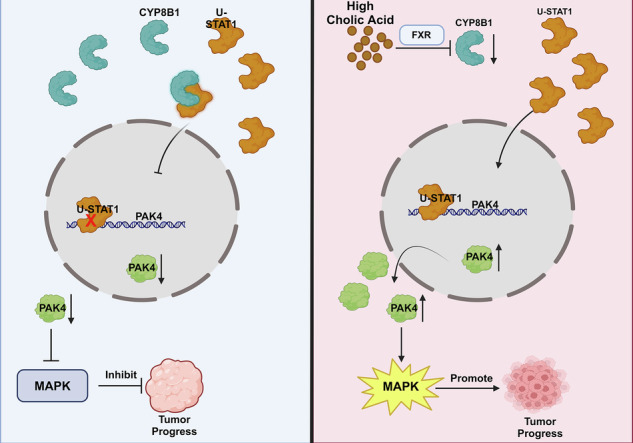

## Introduction

Hepatocellular carcinoma (HCC) is the fourth most commonly diagnosed cancer and the second leading cause of cancer-related deaths in China [1]. Serum metabolomic studies have revealed that the dysregulation of bile acid (BA) metabolism is involved in HCC and chronic liver disease [2]. A large-scale, multicenter study identifying serum metabolite biomarkers in HCC showed significantly elevated levels of most BAs in patients with liver disease compared with those in healthy controls [3, 4]. Elevated circulating levels of BAs are usually caused by increased BA synthesis or hepatic injury [5, 6]. In addition to their key role in fat digestion and vitamin metabolism, BAs are critical signaling molecules that regulate gene expression by targeting nuclear (e.g., FXR) and membrane (e.g., TGR5) receptors, and they have been linked to Non-alcoholic Steatohepatitis(NASH) progression and HCC promotion [7]. BAs downregulate cytochrome P450 8B1 (CYP8B1) expression via FXR and TGR5 in multiple diseases [8–10]. However, mechanistically, how dysregulated BA levels fuel HCC development remains poorly understood.

CYP8B1 is a cytochrome P450 monooxygenase involved in primary BA biosynthesis. BAs are products of cholesterol catabolism, accounting for ~50% of the body’s daily cholesterol turnover, and act as surfactants to facilitate intestinal lipid absorption [11]. The major products of the human BA biosynthetic pathway are cholic acid (CA) and Chenodeoxycholic acid (CDCA) [12]. There are two pathways of BA synthesis: the classical pathway and the alternative pathway, and the classical pathway is predominant in humans. CYP8B1, a sterol 12α-hydroxylase, is thought to play a role in the classical BA synthesis pathway, catalyzing the 12α-hydroxylation of 7α-hydroxy-4-cholesten-3-one to 7α,12α-dihydroxy-4-cholesten-3-one [13]. The balance between these steroids determines the relative levels of CA and CDCA, which in turn determines the hydrophobicity and biological properties of BAs [14]. Thus, CYP8B1 is a key regulator of BA metabolism in humans.

In addition to its involvement in BA metabolism, CYP8B1 is being increasingly recognized as a signaling molecule that activates cell signaling receptors. CYP8B1 aggravates dyslipidemia by activating the ceramide/mTORC1/SREBP-1C pathway via FGF21 and FGF15 [15]. Thus, CYP8B1 may be a useful predictive biomarker for HCC prognosis [16]. Moreover, CYP8B1 may be a potential biomarker for HCC with Metallothionein 1 (MT1) deletion [17]. The metabolic gene *CYP8B1* inhibits esophageal squamous cell carcinoma [18]. Thus, the role and potential mechanisms of action of CYP8B1 in HCC have attracted research attention.

The p21-activated kinases (PAKs) are the major downstream effector proteins of the small Rho GTPases Cdc42 and Rac, which belong to the serine/threonine kinases family [19]. Based on their sequences and functions, the PAK family consists of six members and is categorized into two groups: Group I (PAK1, PAK2, and PAK3) and Group II (PAK4, PAK5, and PAK6) [20]. Among the group II PAKs, PAK4 is the most widely studied member, which regulates a variety of cellular activities, such as cytoskeletal organization, migration, proliferation, survival, and the cell cycle [21]. An increasing number of studies have shown that PAK4 is overexpressed in various tumors and that its dysregulation is associated with cancer progression. PAK4 phosphorylates p53 at serine 215 to promote HCC metastasis [22]. MiRNA-433 inhibits HCC cell proliferation by targeting PAK4 [23] Zic2 promotes tumor growth and metastasis via PAK4 in HCC [24].

Signal transducer and activator of transcription 1 (STAT1) acts as a transcription factor involved in the control of cell differentiation, apoptosis, immune signaling and immune responses [25]. Upon interferon (IFN) stimulation, Janus-activated kinase (JAK) phosphorylates the key tyrosine residue at position 701 of the STAT1 transcription-activating structural domain, resulting in phosphorylated STAT1 (p-STAT1) [26]. p-STAT1 forms homodimers or heterodimers with other members of the STAT family (e.g., STAT3) and then translocates from the cytoplasm to the nucleus, where it functions as a transcription factor. These complexes bind to IFN-stimulated response element (ISRE) and IFN-γ activation sequence (GAS) after translocation to the nucleus [27]. p-STAT1 shifts the dimer conformation from a predominantly antiparallel orientation to a parallel dimer, whereas the unphosphorylated protein (u-STAT1) can exist as a monomer or antiparallel dimer [28]. p-STAT1 is a key activator of IFN signaling, whereas u-STAT1 regulates gene transcription in the absence of IFN stimulation [29, 30]. Thus, p-STAT1 and u-STAT1 can stimulate the transcription of different gene subsets [31]. p-STAT1 regulates the transcription of pro-apoptotic and pro-survival genes to exert tumor suppressor effects in many cancers [32, 33]. u-STAT1 inhibits the expression of Fas and Bad and confers apoptosis resistance to promote the development of sarcoma, whereas pSTAT1 is a tumor suppressor [34]. u-STAT1 is significantly elevated in the tumor tissues of patients with HCC and is mainly expressed in the cytoplasm, whereas p-STAT1 is absent. The loss of u-STAT1 results in significant arrest of the cell cycle and suppressed cell growth in HCC cells [35].

In this study, we analyzed the expression levels of CYP8B1 and PAK4 and their prognostic significance in a cohort of 75 patients with HCC to determine the role of CYP8B1 in HCC cell proliferation and migration and the underlying mechanism and to examine the relationship between CYP8B1 and PAK4.

## Materials and methods

### Cell lines and cell culture

The HUH7 and MHCC-97H cell lines were purchased from the Shanghai Cell Bank of the Chinese Academy of Sciences (CBTCC). The sorafenib-resistant Huh7 cell line (Huh7-SR) was purchased from ORiCells Biotechnology Co., Ltd (Shanghai,China). The cells were cultured in high-glucose DMEM (Hyclone, Logan, UT, USA) supplemented with 10% FBS under standard conditions of 37 °C and 5% CO_2_.

### Tissue samples

Eleven pairs of HCC tissues and adjacent non-tumor tissues were obtained from patients with HCC treated at the Renmin Hospital of Wuhan University from June 2023 to September 2024.

Inclusion Criteria: 1. Adult population aged 18 years and older, 2. Pathohistological results indicated HCC with noncancerous tissues >3 cm away from the cancerous tissues. Tissues were stored in liquid nitrogen. Written informed consent was obtained from all the patients. This study was approved by the Clinical Research Ethics Committee of Hubei Provincial People’s Hospital (Ethics No. 2018 K-G075(Y02)).

Human liver tissue microarray (#HLiv030PG05) was purchased from Shanghai Otto Biotechnology Company (Shanghai, China). The clinical information of the tissue microarrays is in Table S1. Informed consent was obtained from all patients (Ethics No. SHYJS-CP-230701).

### Transfection

Stable overexpression cell lines of CYP8B1 were constructed. Lentivirus of CYP8B1 was purchased from GeneChem (Shanghai, China). Huh7 and 97H cells were infected with the virus solution and screened for puromycin after 24 h. PAK4 was constructed as above.

### Cell proliferation detection by CCK-8 and EdU assays

4000 cells were seeded into 96-well plates, and CCK-8 reagent (Servicebio, Wuhan, China) was added at 0 h, 24 h, 48 h, and 72 h. After incubation for 2 hours in a 37 °C incubator, the absorbance was measured using a microplate reader.

10,000 cells were seeded into 24-well plates. After incubating for 24 hours at 37 °C, EDU reagent was added. Following a 4-hour incubation in the dark, the medium was discarded, and the cells were fixed with 4% paraformaldehyde. The slides were mounted using a DAPI-containing mounting medium, and images were captured using a confocal microscope (Olympus BX51).

### Transwell

30,000 cells were seeded into cell chambers, and Matrigel solution (Corning) was pre-coated for the invasion assay. After 72 hours, the cells were fixed with 4% paraformaldehyde and washed three times with PBS. The cells were then stained with crystal violet (Google Biotech) for 30 minutes. Cells remaining inside the cell chambers were gently wiped off with a damp cotton swab. An inverted microscope was used to capture images.

### Cell cycle experiment

200,000 cells were seeded into six-well plates and incubated at 37 °C for 24 hours before cell cycle analysis. The detailed procedure was carried out according to the instructions provided in the experiment manual (MultiSciences, Hangzhou, China).

### Western blotting

Cells were lysed using RIPA buffer, and the lysates were collected. The samples were then incubated on ice for 30 minutes, followed by the addition of loading buffer and heating at 100 °C in a metal bath for 10 minutes. The lysates were separated by SDS-PAGE and transferred onto PVDF membranes (Bio-Rad). GAPDH was used as the internal control. Band intensities were quantified using ImageLab software. The primary antibodies used were as follows: CYP8B1 (Abmart, TP71907S, 1:750), PAK4 (Proteintech, 14685-1-AP, 1:2000), P-erk1/2 (Santa Cruz, sc-81492, 1:200), erk1/2(Proteintech, 11257-1-AP, 1:8000), P-raf1 (Abclonal, AP0498, 1:2000), Raf-1 (Proteintech, 26863-1-AP, 1:1000), P-mek1/2 (Abclonal, AP1349, 1:2000), mek1/2 (Proteintech, 11049-1-AP, 1:20000), STAT1 (Abclonal, A19563, 1:1000), p-stat1 (Abclonal, AP0054, 1:1000), U-stat1(Abclonal, Cell Signaling Technology, 1:2000) and GAPDH (Proteintech, 60004-1-Ig, 1:100,000), TGR5 (Abclonal, A20778, 1:750), FXR (Abclonal, A24015, 1:1500).

### Bioinformatics

Data of patients with HCC were downloaded from the UCSC Xena website. Four datasets were downloaded from the gene expression omnibus (GEO) database. Finally, they were analyzed in R 4.3.2.

### In vivo growth assays

For the subcutaneous tumor model, male BALB/C nude mice (4 weeks old) were randomly divided into 4/5 groups. Stabilized overexpressed CYP8B1 or control cells were subcutaneously injected into nude mice.

For the lung metastasis model, male BALB/C nude mice (4 weeks old) were randomly divided into four groups. 1 × 10⁶ lentivirus-infected cells were administered via tail vein injection. After three weeks, metastasis status was assessed using in vivo imaging and haematoxylin and eosin (HE) staining.

For the orthotopic liver tumor model, male BALB/C nude mice (4 weeks old) were randomly divided into five groups. Following anesthesia, the liver region was dissected, and 1 × 10⁶ lentivirus-infected cells were injected into the left hepatic lobe. Four weeks later, growth status was assessed via in vivo imaging and HE staining.

All animal experiments were performed in accordance with the guidelines of the Animal Experimentation Center of the Wuhan University People’s Hospital (IACUC Issue No. WDRM 20240403 A).

### Co-immunoprecipitation (Co-IP)

Huh7 cells were collected and lysed in IP lysate, centrifuged and incubated overnight at 4 °C with 5 μg of primary antibodies and protein G agarose (sc-2003, Santa Cruz Biotechnology, USA). Agarose beads were collected by centrifugation at 3000 rpm and washed three times with IP lysate. A 50 μl loading buffer was added, followed by cooking at 100 °C for 10 min. Subsequently, WB validation was performed and 40 μl of IP lysate was taken for liquid chromatography-mass spectrometry analysis.

### Quantitative real-time RT-PCR (qRT-PCR)

Total RNA was extracted from cell or tissue samples using TRIzol reagent (Invitrogen). RNA (1000 ng) was reverse transcribed to cDNA using a One-Step Reverse Transcription Kit (Servicebio). GAPDH was used as an internal control. The sequences of the primers are shown in Table S2, and the relative expression of the target genes was analyzed by the 2^−ΔΔCt^ method.

### Chromatin immunoprecipitation

Assay was performed using the ChIP assay kit (Cell Signaling Technology, USA). PCR amplification was performed using primers targeting specific regions upstream of the PAK4 gene (Table S3).

### Statistical analysis

Measurements between the two groups were analyzed using a two-tailed Student’s *t* test with IBM SPSS Statistics 23 (IBM Corp., USA). A one-way analysis of variance was used to compare the differences between multiple groups. *p* values less than 0.05 were defined as significant (*P < 0.05, **P < 0.01, ***P < 0.001, ****P < 0.0001). Data are expressed as mean ± S.D. of at least three independent experiments. The remaining graphs and curves were plotted using GraphPad Prism 9 software (GraphPad Software, CA, USA).

## Results

### CYP8B1 was downregulated in HCC tissues and predicted a better prognosis

**Fig. 1:**
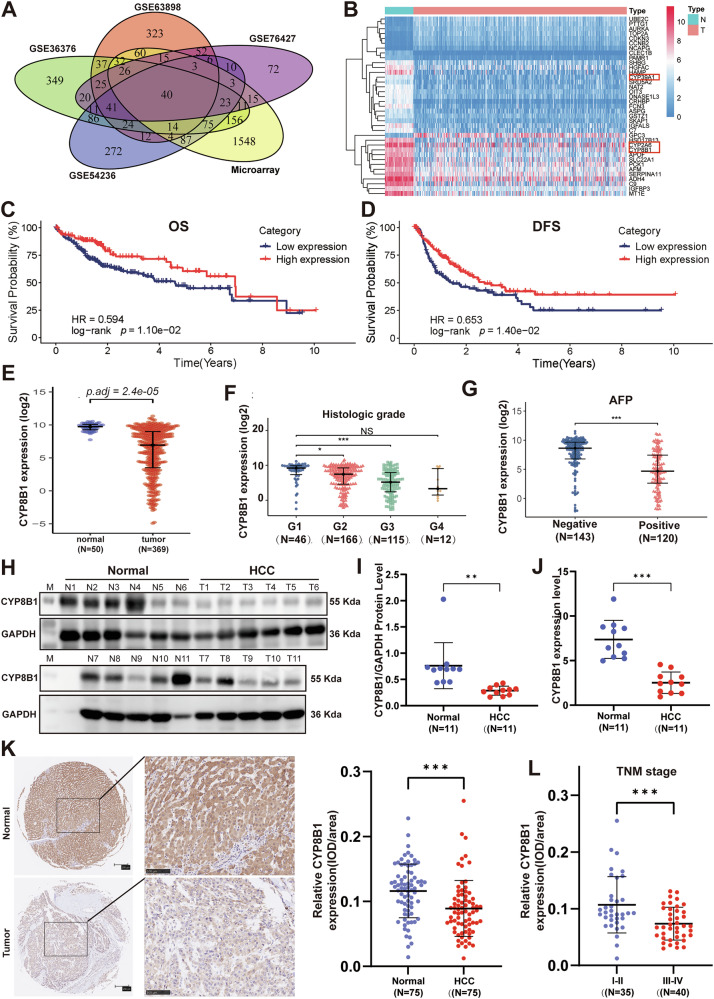
CYP8B1 expression is low in HCC tissues and is associated with good prognosis. **A** Analysis of differential genes from microarray chips and GSE datasets (GSE63898, GSE54236, GSE36376, GSE76427), followed by intersection identification. **B** Heat map of 40 highly variable genes based on TCGA database. **C**, **D** Survival curves indicate the overall and disease-free survival of 324 HCC patients with high or low levels of CYP8B1 expression in TCGA-LIHC, log-rank test. **E** Dot density plot based on TCGA database illustrating the expression of CYP8B1 mRNA in hepatocellular carcinoma and normal liver tissues. **F**,**G** Dot density plots illustrating the mRNA expression of CYP8B1 in different groups of HCC tissues based on the TCGA database. **H**–**J** WB and QPCR analysis of CYP8B1 protein and mRNA levels in 11 pairs of tumor and adjacent-normal tissues. **K** Immunohistochemistry (IHC) staining of tissue microarrays from 75 HCC patients, with calculation of average optical density values. Dot density plots indicate the levels of CYP8B1 protein in tumor and adjacent-normal tissues in the tissue microarray of 75 patients with hepatocellular carcinoma. **L** Dot density plot illustrating the expression of CYP8B1 in hepatocellular carcinoma and adjacent-normal tissues. *P < 0.05, **P < 0.01, ***P < 0.001, NS not significant.

We identified the differentially expressed genes in the HCC microarray chip from our study group and the four GSE datasets and obtained 40 differentially expressed genes (Fig. 1A). Then, we generated a heat map of these 40 genes using the TCGA database (Fig. 1B). We found that patients with HCC exhibiting high expression of CYP8B1 had longer overall and progression-free survival times (Fig. 1C, D). CYP8B1 was expressed at low levels in HCC tissues (369 tumors, 50 normal; Fig. 1E), and CYP8B1 was correlated with pathological stage and AFP expression. Higher pathological stages were associated with lower CYP8B1 expression in G1, G2, and G3 patients with HCC; patients with G4 stage HCC also showed this trend, but the difference was not statistically significant due to the small number of cases (Fig. 1F). CYP8B1 expression was low in patients with HCC and elevated AFP levels (Fig. 1G). Subsequently, we examined the expression levels of CYP8B1 in tumor and non-tumor tissues from 11 pairs of HCC patients using western blotting (WB) and quantitative polymerase chain reaction (qPCR), which showed that the mRNA and protein levels of CYP8B1 in tumor tissues were significantly lower than those in non-tumor tissues (Fig. 1H–J). We further examined the expression of CYP8B1 in tissue microarrays containing 75 pairs of HCC samples by IHC, which also demonstrated that the expression of CYP8B1 in tumor tissues was lower than that in adjacent non-tumor tissues (Fig. 1K). CYP8B1 expression was lower in pTNM stage III-IV patients than in stage I–II patients (*P* < 0.0001), and postoperative pathology showed that CYP8B1 expression was lower in patients with vascular invasion (*P* = 0.0017) (Fig. 1L). This implies that CYP8B1 expression predicts a better prognosis in patients.

### Cholic acid promoted proliferation and migration in HCC cells via CYP8B1

**Fig. 2:**
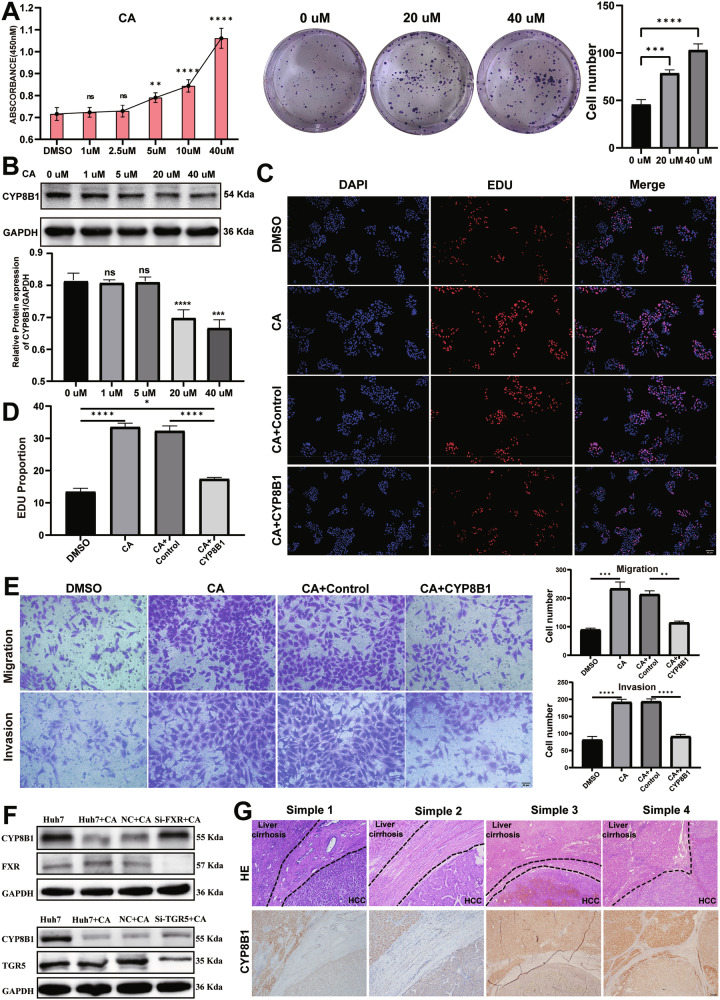
CA promotes hepatocellular carcinoma progression by downregulating CYP8B1. **A** CCK-8 assay and Colony formation assay to detect the effects of CA at different concentrations on the proliferation of hepatocellular carcinoma cell line (Huh7). **B** WB detection of CYP8B1 protein expression in hepatocellular carcinoma cells cultured with different concentrations of CA. **C**, **D** EDU experiment illustrates that CA promotes the proliferation of hepatocellular carcinoma cells, which is reversed by overexpression of CYP8B1. **E** Transwell assay illustrates that CA promotes invasion and migration of hepatocellular carcinoma cells, which is reversed by overexpression of CYP8B1. **F** Detection of FXR, TGR5 and CYP8B1 expression levels by western blot. **G** Protein expression level of CYP8B1 in four cases of hepatocellular carcinoma and severe cirrhosis was detected by IHC. Scale: 200 μm. **P < 0.01, ***P < 0.001, ****P < 0.0001, *NS* not significant.

BAs in the human body consist mainly of cholic acid (CA) and CDCA. Obeticholic acid (OCA) inhibits the proliferation and metastasis of HCC cells [36]. CCK-8 and colony formation assays showed that treatment of Huh7 cells with different concentrations of CA promoted cell proliferation, and the effect was concentration-dependent. However, the treatment of Huh7 cells with different concentrations of CDCA and OCA for 3 days inhibited cell proliferation in a concentration-dependent manner (Figs. 2A and S1A). WB showed that CA inhibited the expression of CYP8B1 in Huh7 cells, and this inhibitory effect was concentration-dependent (Fig. 2B). EdU and Transwell experiments showed that CA promoted the proliferation and migration of Huh7 cells, and the overexpression of CYP8B1 reversed this pro-carcinogenic phenomenon (Fig. 2C–E). FXR and TGR5 are the primary receptors of CA [37]. To investigate whether CA regulates the expression of CYP8B1 through FXR and TGR5 in HCC, we knocked down FXR and TGR5 individually (Fig. S1B). The experimental results showed that knocking down FXR attenuated the inhibitory effect of CA on CYP8B1, whereas the reversal effect of knocking down TGR5 was relatively weaker (Fig. 2F). To further verify whether the expression of CYP8B1 in HCC is primarily regulated by high levels of CA, we further examined the expression of CYP8B1 in the tissues of four patients with HCC and cirrhosis using IHC. The results also showed that the expression of CYP8B1 in tumor tissues was lower than that in adjacent non-tumor tissues (with liver cirrhosis) (Fig. 2G). These findings indicate that the low expression of CYP8B1 in HCC is partially caused by CA.

### CYP8B1 inhibited proliferation and migration in HCC cells

**Fig. 3:**
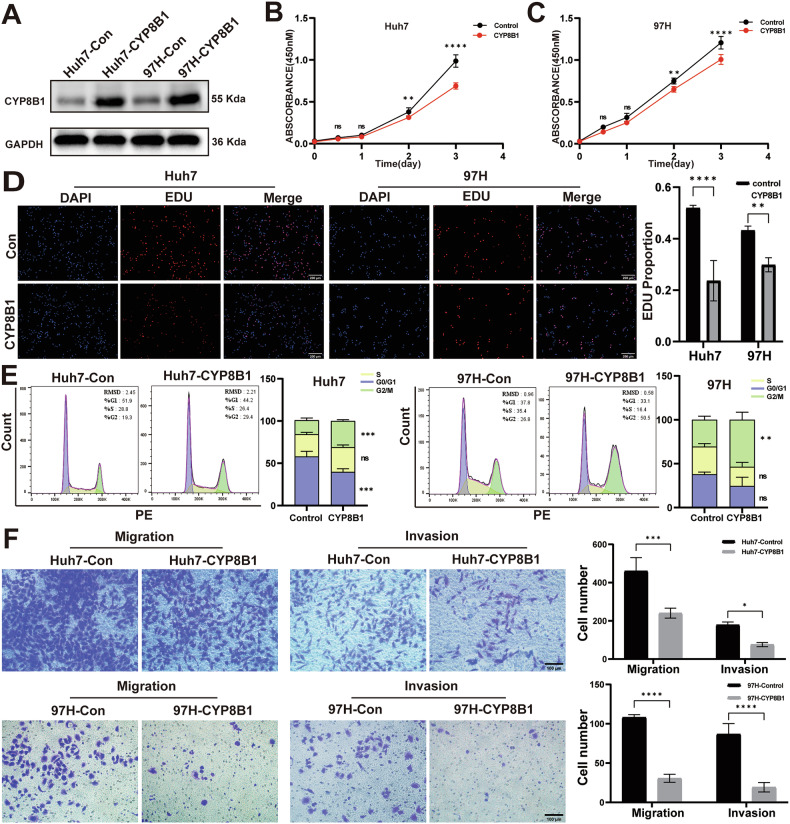
CYP8B1 inhibits the progression of hepatocellular carcinoma. **A** The efficiency of CYP8B1 overexpression was detected by WB. **B**, **C** The effects of CYP8B1 on cell proliferation were determined by CCK-8 assays using cells with CYP8B1 overexpression. **D** The effects of CYP8B1 on cell proliferation were determined by EDU assays using cells with CYP8B1 overexpression. Scale: 200 μm. **E** The effects of CYP8B1 on the cell cycle were determined by flow cytometry. **F** The effects of CYP8B1 on cell invasion and migration were determined by Transwell assay. Scale: 100 μm. *P < 0.05, **P < 0.01, *** *P* < 0.001, ****P < 0.0001, *NS* not significant.

To validate the biological function of CYP8B1 in HCC, we generated Huh7 and MHCC-97H cells stably overexpressing CYP8B1 and verified their transfection efficiency using WB (Fig. 3A). CCK-8 and EdU assays showed that the proliferation of Huh7 and MHCC-97H cells overexpressing CYP8B1 was reduced (Fig. 3B–D). Flow cytometry experiments showed that Huh7 and MHCC-97H cells overexpressing CYP8B1 exhibited a G2/M phase block (Fig. 3E). Transwell experiments showed that Huh7 and MHCC-97H cells overexpressing CYP8B1 exhibited significantly reduced invasion and migration (Fig. 3F). In conclusion, CYP8B1 inhibited the proliferation and migration of HCC cells.

### CYP8B1 suppresses PAK4 expression and inhibits MAPK signaling

**Fig. 4:**
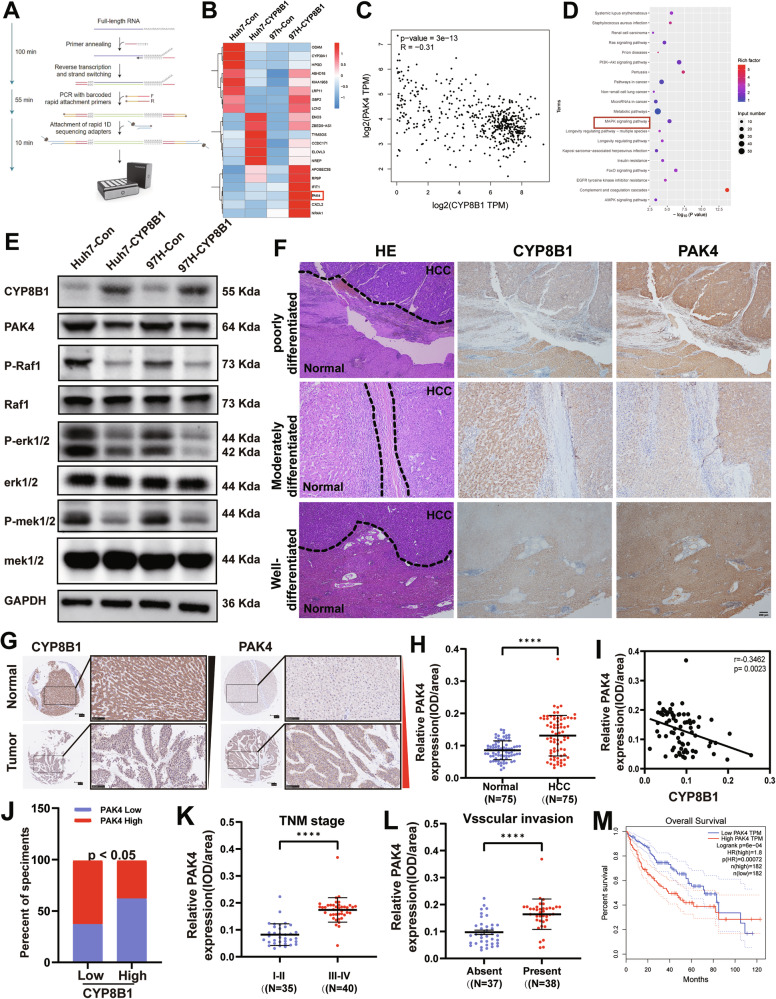
CYP8B1 suppresses PAK4 expression and inhibits MAPK signaling. **A** Flow chart of Nanopore long-read RNA-sequencing analysis. **B** Heat map of 20 differential genes. **C** Correlation analysis between CYP8B1 and PAK4 based on GEPIA database. **D** KEGG enrichment analysis based on Nanopore long-read RNA-sequencing analysis. **E** The expression levels of CYP8B1, PAK4, p-MEK, MEK, p-Raf, RAF, p-ERK and ERK were examined by western blotting. **F** Representative HE and IHC staining results of CYP8B1 and PAK4 in differently differentiated tumor and non-tumor tissues are shown. Scale: 200 μm. **G** The expression of CYP8B1 and PAK4 in 75 pairs of HCC tissues was examined by IHC. **H** Protein expression of PAK4 was higher in tumor tissues compared to adjacent normal tissues. **I**, **J** Negative correlation between protein expression of CYP8B1 and PAK4 based on tissue microarrays. All *P* < 0.05. **K**, **L** Dot density plots illustrate the expression of CYP8B1 in different groups of HCC tissues. **M** Survival curves indicate the overall survival of 324 HCC patients with high or low levels of CYP8B1 expression in TCGA-LIHC, log-rank test. ****P < 0.0001.

Nanopore long-read RNA-sequencing analysis was performed to determine the underlying mechanism of CYP8B1 in HCC. We analyzed the differential expression of mRNA in HCC cells overexpressing CYP8B1 and control HCC cells (Fig. 4A). Using a threshold of |log2FC | >1.5, we identified 20 genes (Fig. 4B). According to the GEPIA database, CYP8B1 was negatively correlated with PAK4 (*R* = −0.31, *p* = 3e-13; Fig. 4C). KEGG pathway enrichment analysis of the sequencing results of Huh7 overexpressing CYP8B1 and the controls suggested CYP8B1 overexpression may affect the MAPK signaling pathway (Fig. 4D). WB revealed that CYP8B1 overexpression in Huh7 and MHCC-97H cells inhibited the expression of PAK4, whereas CYP8B1 overexpression inhibited the expression of the phosphorylated forms of Raf, MEK, and ERK (Fig. 4E). The expression levels of CYP8B1 and PAK4 were detected using IHC in highly, moderately, and poorly differentiated HCC tissues, respectively, and it was found that CYP8B1 expression was higher in paracancerous tissues than in HCC tissues, and PAK4 expression was lower in paracancerous tissues than in HCC tissues, and the difference in PAK4 expression was more pronounced in the more poorly differentiated HCCs (Fig. 4F). Together, these findings support a potential role of CYP8B1 in repressing MAPK signaling via PAK4.

### CYP8B1 and PAK4 expression are correlated in clinical HCC tissues, and PAK4 predicts poor prognosis

PAK4 is regulated by CYP8B1 in HCC cells; therefore, its relationship with clinical tissues was investigated. IHC analysis of PAK4 in tissue microarrays containing 75 pairs of HCC tissues, combined with the previous CYP8B1 staining results, showed that low expression of CYP8B1 was often accompanied by high expression of PAK4 (Fig. 4G). Correlation analysis of the staining results of the two tissue microarrays revealed that the expression of CYP8B1 and PAK4 was negatively correlated at the protein level (Fig. 4I, J). Further analysis of tissue microarrays for PAK4 showed that its expression was higher in tumor tissues than in adjacent non-tumor tissues (Fig. 4H). We observed that the expression of PAK4 was higher in pTNM stage III-IV patients than in stage I–II patients (*P* < 0.0001; Fig. 4K), and the postoperative pathology results showed that the expression of PAK4 was higher in patients with vascular invasion (*P* < 0.0001; Fig. 4L). Using the TCGA database, we found that patients with HCC and low expression of PAK4 had longer overall survival times compared with patients with high expression of PAK4 (Fig. 4M).

### Overexpression of PAK4 reversed CYP8B1-inhibited cell proliferation and migration in HCC cells

**Fig. 5:**
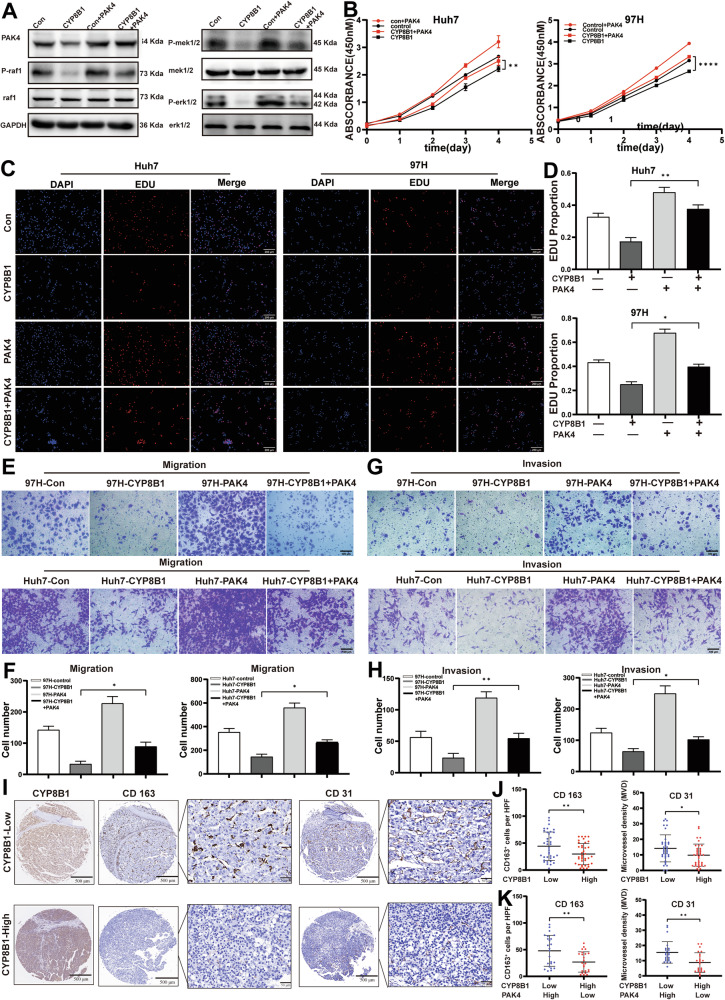
PAK4 reverses the anticancer effect of CYP8B1. **A** The expression levels of PAK4, p-MEK, MEK, p-Raf, RAF, p-ERK and ERK were examined by western blotting. CCK-8 (**B**), EDU (**C**, **D**) and Transwell assay (**E**–**H**) demonstrate that overexpression of PAK4 reverses the anticancer effect of CYP8B1. Scale: 100 μm. **I**–**K** The expression of CD163 and CD31 in 75 pairs of HCC tissues was examined by IHC. *P < 0.05, **P < 0.01, ****P < 0.0001.

The effect of PAK4 on CYP8B1-inhibited proliferation and migration of HCC cells was investigated. WB showed that overexpression of CYP8B1 in Huh7 cells significantly decreased the phosphorylation of Raf, ERK, and MEK, which were increased by PAK4 (Fig. 5A). CCK-8 and Edu assays showed that PAK4 overexpression in Huh7 and MHCC-97H cells significantly enhanced CYP8B1-inhibited cell proliferation (Fig. 5B–D). Transwell experiments showed that the overexpression of PAK4 in Huh7 and MHCC-97H cells significantly increased CYP8B1-inhibited cell migration and invasion (Fig. 5E–H). Subsequently, in a tissue microarray of 75 patients with HCC, we assessed CD31 (an endothelial marker) and CD163 (an M2 macrophage marker) expression by IHC. It was found that CYP8B1 expression significantly inversely correlated with both CD31 and CD163 levels (Fig. 5I, J). Patients with HCC characterized by high CYP8B1 and low PAK4 expression exhibited significantly lower CD163 and CD31 levels compared with those with low CYP8B1 and high PAK4 expression. This difference was more evident than that observed with CYP8B1 expression alone (Fig. 5K). These findings indicate that the CYP8B1/PAK4 axis may play a critical role in modulating HCC metastasis.

### CYP8B1 inhibited tumor proliferation in vivo, PAK4 reversed the inhibitory effect of CYP8B1, and MEK pathway inhibitors reversed the inhibitory effect of PAK4

**Fig. 6:**
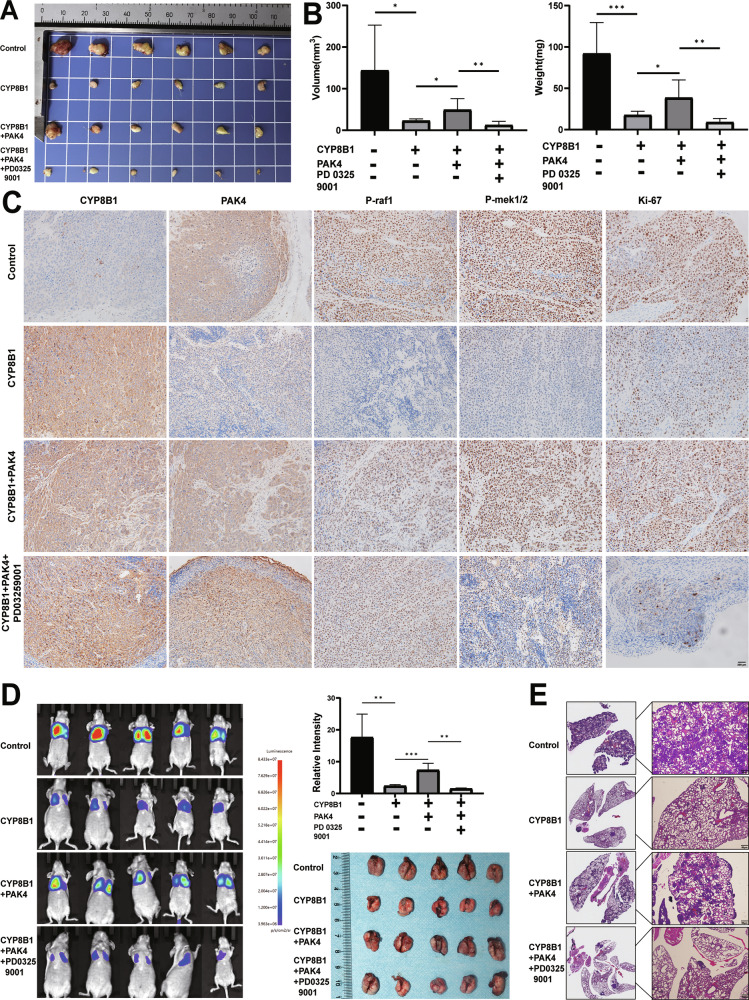
CYP8B1 inhibits tumor proliferation and metastasis in nude mice. **A** Huh7 cell line overexpressing CYP8B1, PAK4, and PAK4 was injected into nude mice. When tumors reach a maximum diameter of 3 mm, mice are treated with PD0325901 (20 mg/kg once daily). At a maximum tumor diameter of 10 mm, the mice were euthanized, and the tumors were removed, and the tumor size and weight (**B**) were measured. **C** Immunohistochemistry (IHC) determined the expression of CYP8B1, PAK4, P-raf1, P-mek1/2 and Ki67 in subcutaneous xenograft tumors. **D** Representative in vivo imaging of a lung metastasis model. **E** Representative HE staining of lung sections in (**D**). Scale: 200 μm. *P < 0.05, **P < 0.01, ***P < 0.001, ****P < 0.0001, *NS* not significant.

In the Huh7-based xenograft model, overexpression of CYP8B1 significantly inhibited tumor growth. When Huh7 cells co-transfected with CYP8B1 and PAK4 were injected into the xenograft model, this inhibitory effect was reversed, and a pro-proliferative phenomenon was demonstrated. And when nude mice injected with Huh7 cells co-transfected with CYP8B1 and PAK4 were treated with PD03259001 (an MEK inhibitor), the pro-proliferative phenomenon was reversed (Fig. 6A, B). Subsequently, IHC analysis of protein expression in tumors showed that expression of PAK4, p-raf1, p-mek1/2, and Ki-67-positive levels were reduced in xenografts overexpressing CYP8B1, and that PAK4 reversed the expression of these proteins, which was in turn reversed by the inhibitor PD03259001(Fig. 6C). We subsequently established a lung metastasis model through tail vein injection. The results demonstrated that CYP8B1 significantly inhibits the lung metastasis of HCC cells, while the overexpression of PAK4 can reverse the anti-metastatic effect of CYP8B1 (Fig. 6D, E). These results suggest that CYP8B1 inhibits cell proliferation and migration, partially by downregulating PAK4 expression. Moreover, CYP8B1 inhibited cell growth and metastasis through the PAK4/MEK/ERK pathway.

### CYP8B1 restores the sensitivity to sorafenib in HCC by regulating PAK4

Sorafenib is a multi-target small-molecule tyrosine kinase inhibitor primarily used for the treatment of advanced HCC, advanced renal cell carcinoma, and certain types of thyroid cancer. It exerts antitumor effects by inhibiting the RAF/MEK/ERK signaling pathway, thereby interfering with tumor cell proliferation and suppressing cell growth. However, resistance to sorafenib significantly compromises the prognosis of patients with HCC [38].

**Fig. 7:**
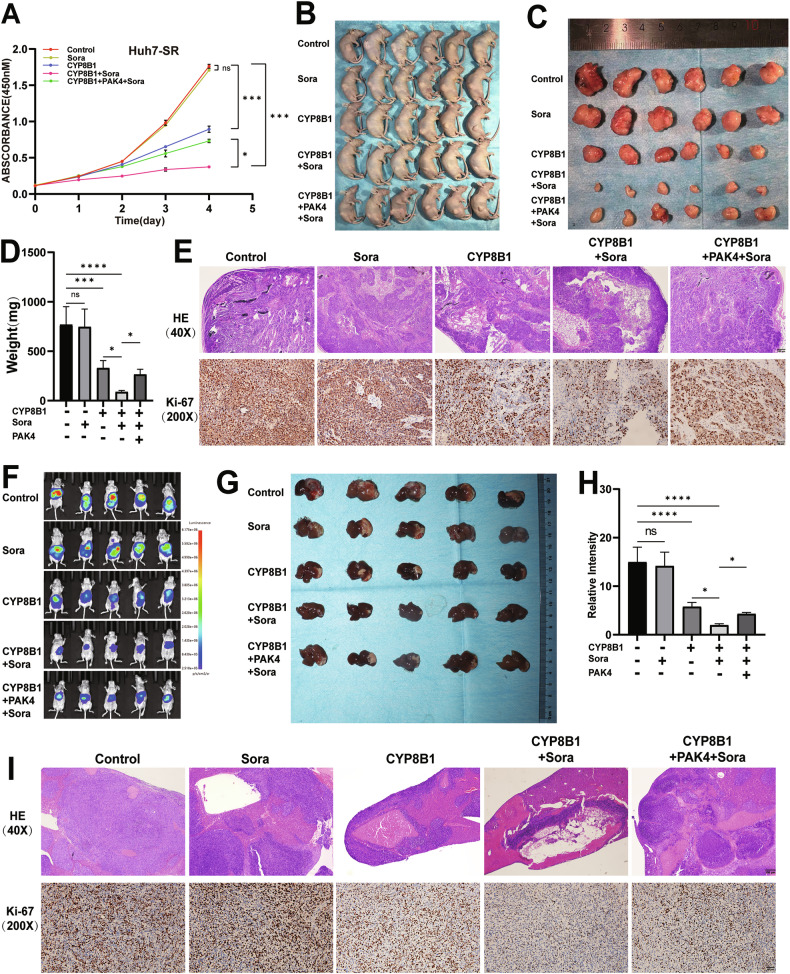
CYP8B1/PAK4 axis restores sensitivity to sorafenib in HCC cells. **A**–**C** Sorafenib combined with CYP8B1 overexpression significantly inhibited the proliferation of sorafenib-resistant cells in vivo and in vitro, whilst PAK4 overexpression reversed the effect of CYP8B1 (*n* = 6). **D** Weight of subcutaneous xenograft tumors. **E** Ki67 expression of subcutaneous xenograft tumors. **F**–**H** Sorafenib combined with CYP8B1 overexpression significantly inhibited the proliferation of sorafenib-resistant cells in the orthotopic tumor model, whilst PAK4 overexpression reversed the effect of CYP8B1. **I** Ki67 expression of orthotopic tumor. Scale: 50 μm. *P < 0.05, **P < 0.01, ***P < 0.001, ****P < 0.0001, *NS* not significant.

This study demonstrated that CYP8B1 can suppress the RAF/MEK/ERK signaling pathway by regulating PAK4. Therefore, we sought to investigate the role of CYP8B1 in sorafenib resistance. By overexpressing CYP8B1 in sorafenib-resistant HCC cell lines (Huh7-SR), we observed that CYP8B1 overexpression enhanced the sensitivity of HCC cells to sorafenib, whereas PAK4 overexpression reversed the effects of CYP8B1 by CCK-8 assay (Figs. 7A and S1C). These findings were further validated in vivo using subcutaneous tumorigenesis experiments in nude mice, which confirmed that the CYP8B1/PAK4 axis regulates sorafenib resistance in HCC cells (Fig. 7B–E). Additional verification using a liver orthotopic tumor model produced consistent results, further reinforcing this conclusion (Fig. 7F–I).

### CYP8B1 specifically bound to u-STAT1 in the cytoplasm, resulting in reduced u-STAT1 translocation to the nucleus and, in turn, reduced PAK4 transcription

**Fig. 8:**
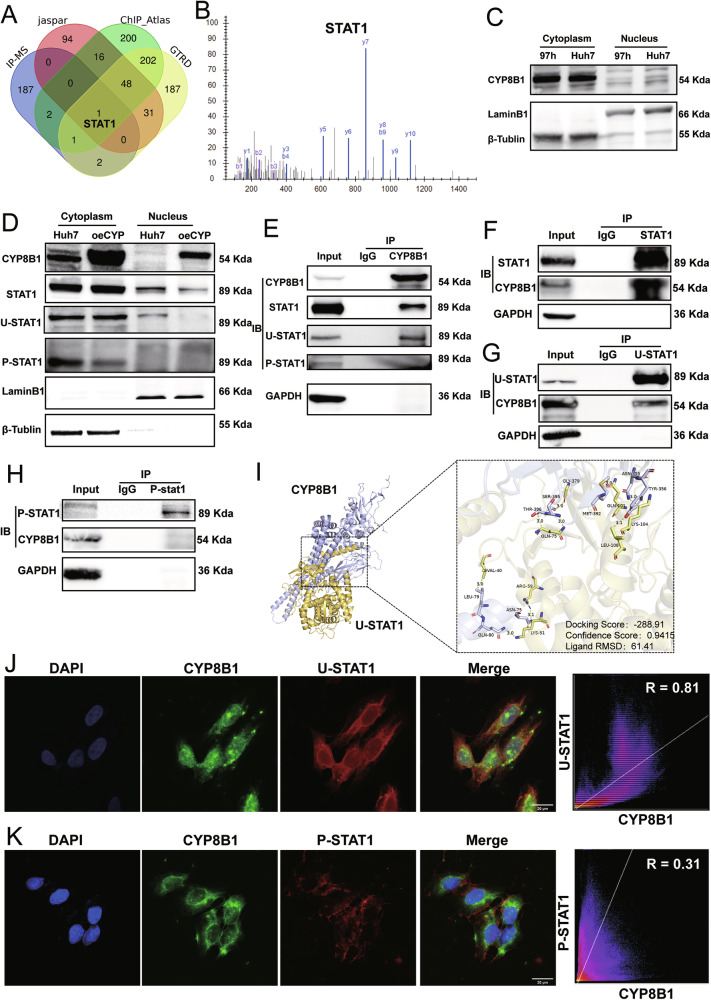
CYP8B1 binds to u-STAT1, thereby inhibiting u-STAT1 entry into the nucleus. **A** The results of IP-MS for CYP8B1, as well as transcription factor prediction for u-STAT1, were shown. **B** The mass spectrometry peaks of STAT1 were shown. **C** Nucleoplasmic separation assays demonstrated that CYP8B1 is predominantly expressed in the cytoplasm. **D** When CYP8B1 was overexpressed, u-STAT1 entry into the nucleus was reduced. CO-IP assays **E**–**H**, Molecular docking assays (**I**) and immunofluorescence colocalization (**J**, **K**) demonstrated that CYP8B1 binds to u-STAT1. Scale: 20 μm.

CYP8B1-binding proteins were identified using immunoprecipitation and mass spectrometry. The mass spectrometry data are presented in Table S4. PAK4-related transcription factors were predicted using the JASPAR, ChIP_Atlas, and GTRD databases (Table S5). STAT1 was identified as the only common protein among the above four databases (Fig. 8A), indicating that STAT1 may specifically bind to CYP8B1 and may also be a transcription factor for PAK4 (Fig. 8B). Nuclear-cytoplasmic separation experiments showed that CYP8B1 was expressed in both the nucleus and cytoplasm of Huh7 and MHCC-97H cells, and was mainly expressed in the cytoplasm (Fig. 8C). Overexpression of CYP8B1 reduced the translocation of u-STAT1 from the cytoplasm to the nucleus but did not affect the nuclear and cytoplasmic distribution of p-STAT1 (Fig. 8D). Co-immunoprecipitation assays showed that there was a protein-protein interaction between CYP8B1 and u-STAT1 in Huh7 cells and that CYP8B1 did not bind to p-STAT1 (Fig. 8E–H). Additionally, we performed molecular docking analysis of CYP8B1 and u-STAT1 (Fig. 8I). Cellular immunofluorescence colocalization assays showed that CYP8B1 and u-STAT1 were strongly colocalized in Huh7 cells (*r* = 0.81; Fig. 8J), whereas the colocalization of CYP8B1 and p-STAT1 was very low (*r* = 0.31; Fig. 8K).

**Fig. 9:**
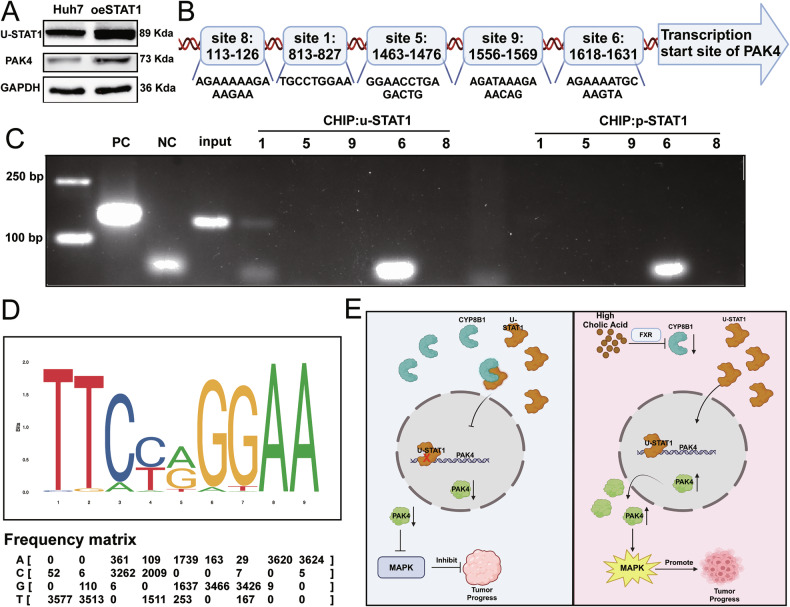
u-STAT1 promotes the transcription of PAK4. **A** WB assay showed that overexpression of u-STAT1 upregulated PAK4. **B** Possible binding sites for PAK4 were predicted by the JASPAR database. **C** CHIP assays demonstrated the binding site of u-STAT1 and PAK4. **D** JASPAR database predicts possible binding sites. **E** Schematic model illustrating the proposed mechanism.

WB showed that in Huh7 and MHCC-97H cells, overexpression of STAT1 can increase the expression of u-STAT1 and the expression of PAK4 (Fig. 9A). We predicted nine promoter sites of PAK4 using the Jaspar database that may bind to the transcription factor u-STAT1 and selected five sites with a score>5 for chromatin immunoprecipitation (ChIP) verification (Fig. 9B). ChIP assays showed that in Huh7 cells, the PAK4 promoter gene site 1 (813–27) binds to u-STAT1, and no promoter site on the PAK4 gene bound to p-STAT1 (Fig. 9C). The possible binding sequence is TTCCAGGAA (Fig. 9D). These results confirmed that u-STAT1 is a transcription factor of PAK4 and can bind to a specific site in the PAK4 promoter sequence. In summary, elevated BA levels inhibit the protein expression of CYP8B1, thereby reducing the binding of u-STAT1. The increased translocation of u-STAT1 from the cytoplasm to the nucleus promotes the transcription of PAK4, thereby activating the MAPK pathway. This promotes tumorigenesis (Fig. 9E).

## Discussion

Studies in clinical patients and animal models have shown that abnormal BA levels are associated with liver diseases, such as HCC [39]. Elevated levels of primary and secondary BAs, as well as elevated levels of BAs metabolism, have been detected in patients with HCC [3, 4]. BAs are critical signaling molecules that regulate CYP8B1 expression by targeting nuclear (e.g., FXR) and membrane (e.g., TGR5) receptors [7, 8]. BAs in the human body consist mainly of CA and CDCA. CYP8B1 is a sterol 12α-hydroxylase involved in CA synthesis during normal biological activity. This study found that CYP8B1 inhibits the development of HCC and that CA promotes the proliferation and migration of Huh7 cells by inhibiting CYP8B1, which further clarifies the mechanism by which BAs promote cancer. Cirrhosis and HCC cause cholestasis and liver damage, and BAs can inhibit CYP8B1 expression. However, in this study, in four cases of liver cirrhosis with HCC, IHC staining still revealed that the expression of CYP8B1 in liver cirrhosis tissue was higher than that in HCC tissue. This indicates that downregulated CYP8B1 expression in HCC tissues is not exclusively caused by an increase in BA level.

We identified 40 differentially expressed genes by analyzing our group’s microarray chip and four GSE databases. These differentially expressed genes were analyzed using the TCGA database to obtain CYP8B1. Further survival analysis of CYP8B1 using TCGA showed that CYP8B1 was associated with a good prognosis. In addition, tissue microarrays containing 75 pairs of HCC samples showed that CYP8B1 expression was lower in HCC tissues than in normal tissues, and that the higher the pathological grade, the lower the expression of CYP8B1. CYP8B1 expression was also lower in HCC tissues with elevated AFP levels, indicating that CYP8B1 is associated with good prognosis. In summary, clinical data confirmed that CYP8B1 is expressed at low levels in HCC tissues and is associated with good prognosis. In vivo and in vitro experiments showed that CYP8B1 inhibited the proliferation and migration of HCC cells.

Next, we identified 20 differentially expressed genes and signaling pathways by performing nanopore long-read RNA-sequencing analysis of CYP8B1-overexpressing HCC cells, among which PAK4 was closely related to CYP8B1 and the MAPK pathway was also related to CYP8B1. WB confirmed that CYP8B1 inhibited the expression of PAK4 and phosphorylation of the Raf/MEK/ERK pathway. Many studies have shown that PAK4 promotes HCC cell growth by regulating the phosphorylation of the Raf/MEK/ERK pathway [24, 40]. In this study, tissue microarrays were used to verify the strong correlation between CYP8B1 and PAK4 expression. In vitro and vivo studies showed that PAK4 reversed the inhibition of HCC cell proliferation and migration induced by CYP8B1. Sorafenib is a multi-kinase inhibitor that promotes apoptosis, reduces angiogenesis, and suppresses tumor cell proliferation. It is an effective first-line treatment for advanced HCC. However, the emergence of sorafenib resistance has become increasingly common [38]. Sorafenib inhibits tumor cell proliferation by targeting kinases such as Raf-1, B-Raf, and those within the Ras/Raf/MEK/ERK signaling pathway [41]. PAK4 is a key activator of the classical MAPK signaling pathway [19]. In this study, we demonstrated that CYP8B1 enhances the sensitivity of HCC cells to sorafenib by regulating PAK4. Through IP-MS analysis of CYP8B1 and transcription factor prediction of PAK4, we identified STAT1, which may directly bind to CYP8B1 and act as a potential transcription factor for PAK4. ChIP confirmed that u-STAT1 binds directly to the PAK4 promoter. CYP8B1 binds to u-STAT1 in the cytoplasm and reduces its translocation to the nucleus, thereby inhibiting the transcription of PAK4 and ultimately inhibiting the phosphorylation of the Raf/MEK/ERK pathway.

In summary, our findings indicate that the newly discovered CYP8B1/PAK4 axis plays an important role in HCC progression and further elucidates the mechanism by which BAs promote HCC. Thus, CYP8B1 is a potential therapeutic target for the clinical treatment of HCC.

## Supplementary information

Figure S1

TableS1

TableS2

TableS3

TableS4

TableS5

Supplementary Material Legends

Uncropped western blots

STR

## Data Availability

The datasets used and/or analyzed during the current study are available from the corresponding author on reasonable request. The RNA-seq datasets generated in this study have been deposited in the NCBI Sequence Read Archive (SRA) under the accession number PRJNA1334110.
