# Supplementary figures and images for: CYP8B1 inhibits hepatocellular carcinoma progression by repressing PAK4 transcription through inhibition of nuclear translocation of u-STAT1
Source: Cell Death Dis. 2025 Dec 31;17(1):172. doi: 10.1038/s41419-025-08393-3 (PMC12876879; doi:10.1038/s41419-025-08393-3)

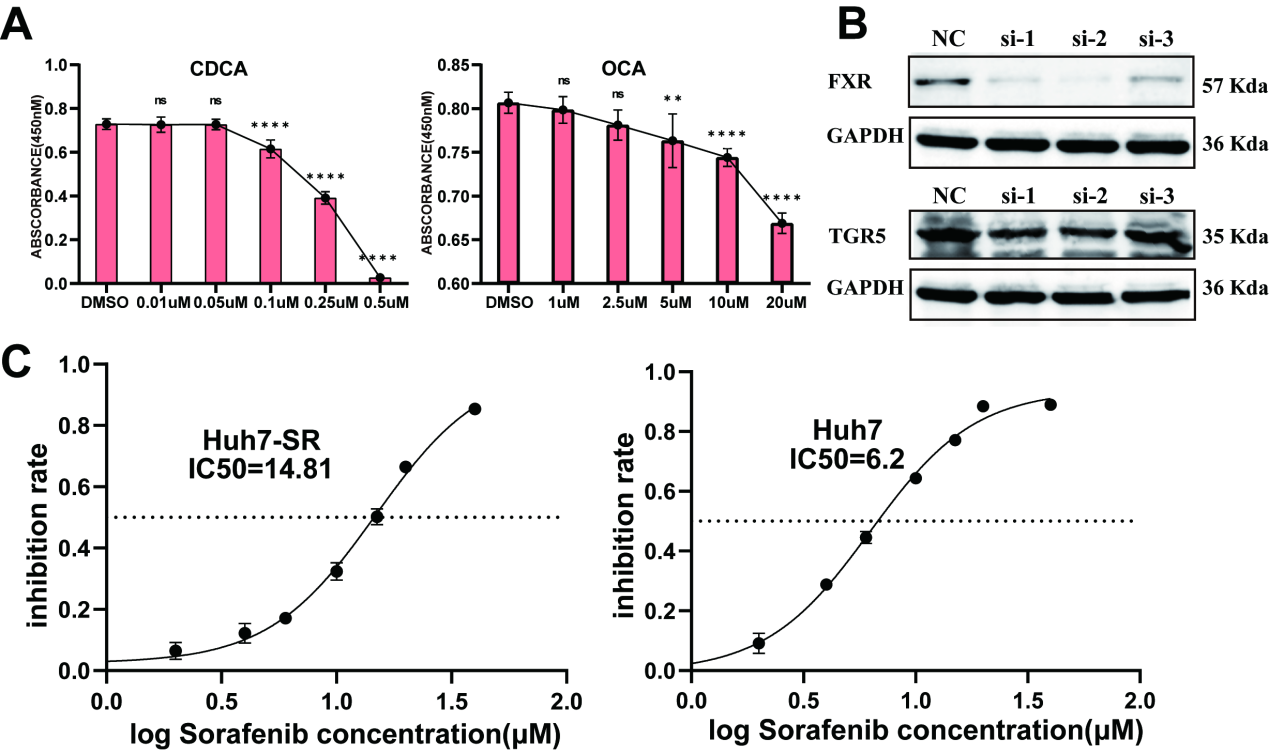

Supplement: Supplementary file 1 — Figure S1 [file 41419_2025_8393_MOESM1_ESM.docx]

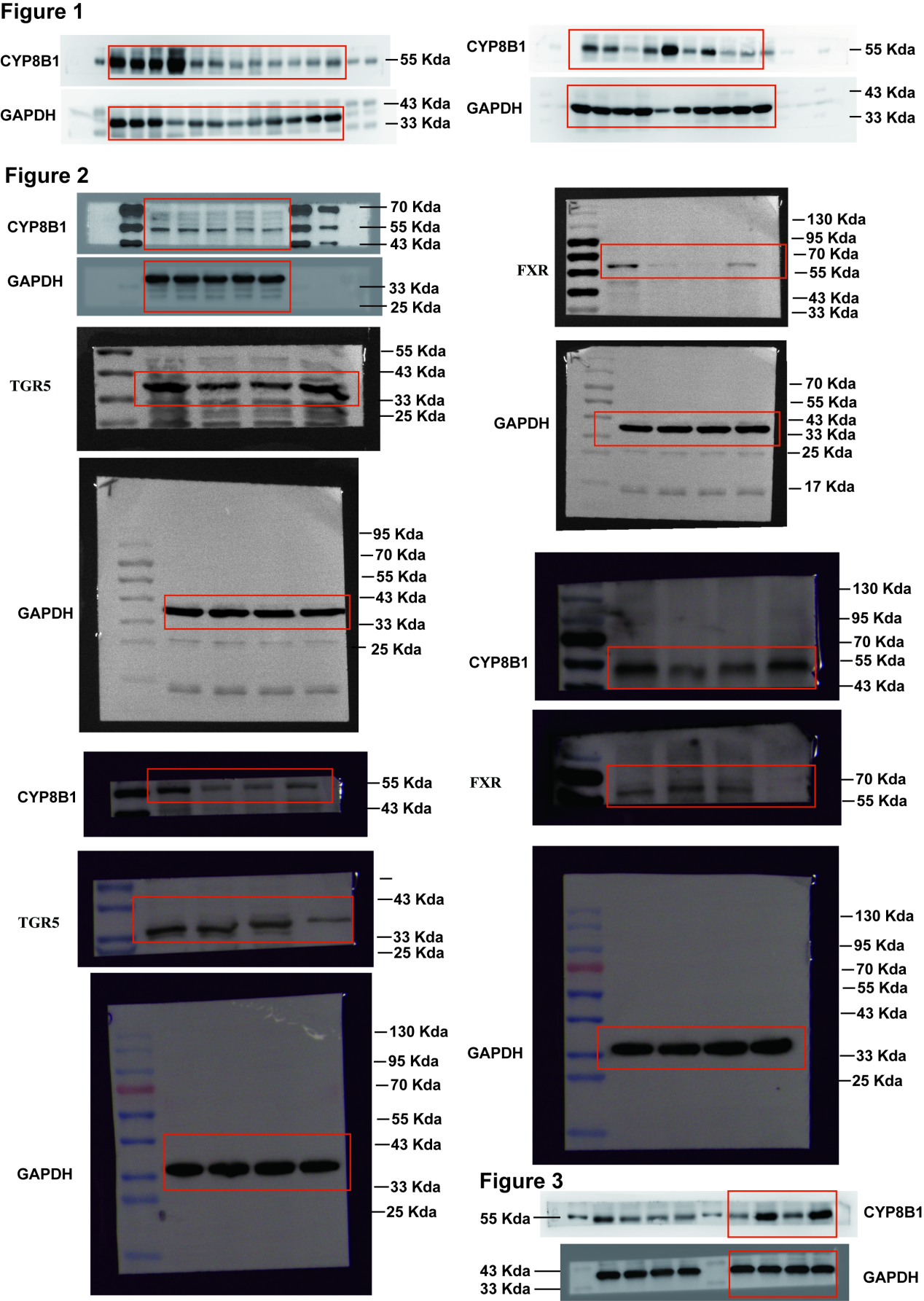

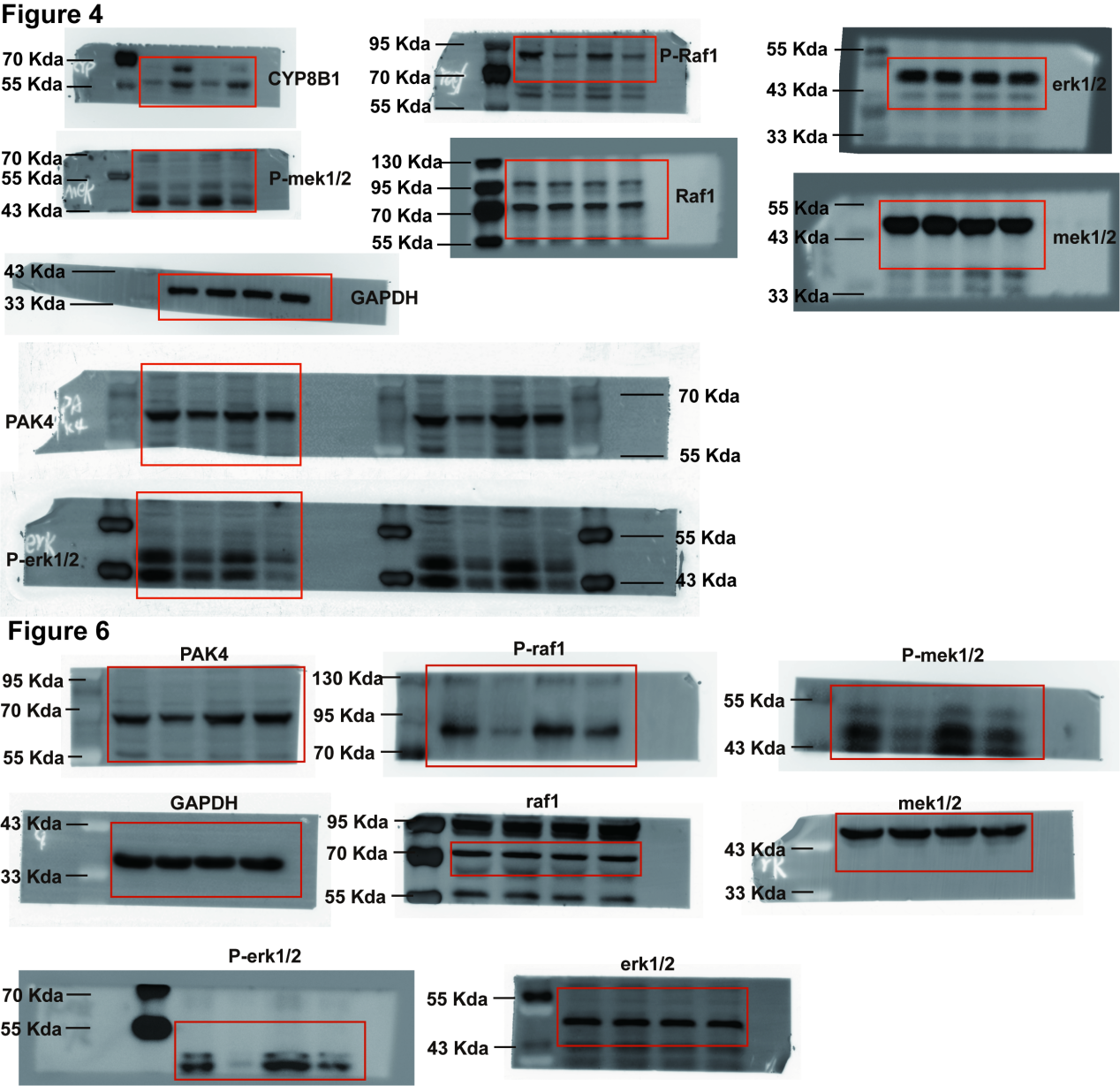

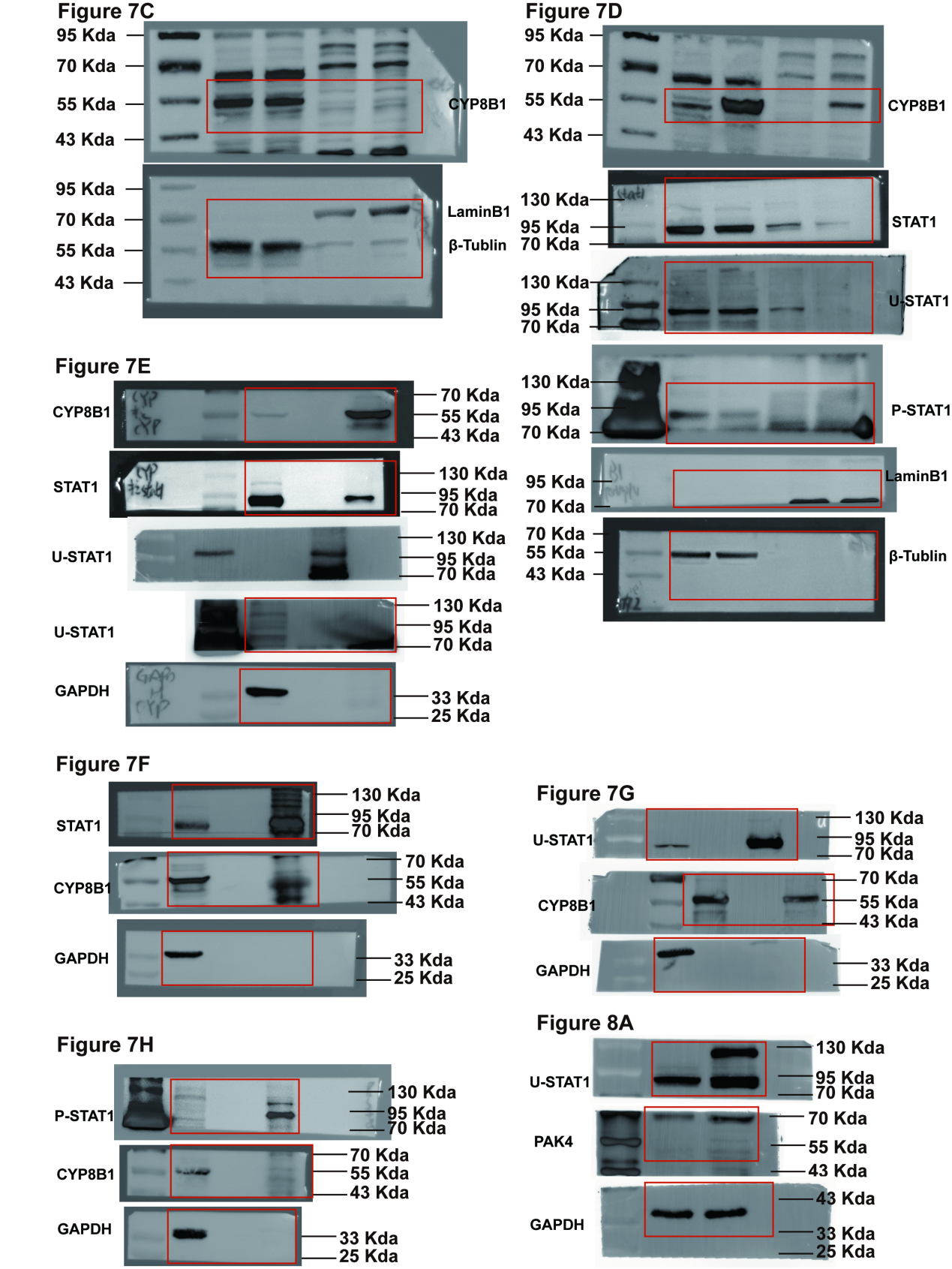

Supplement: Supplementary file 8 — Uncropped western blots [file 41419_2025_8393_MOESM8_ESM.docx]

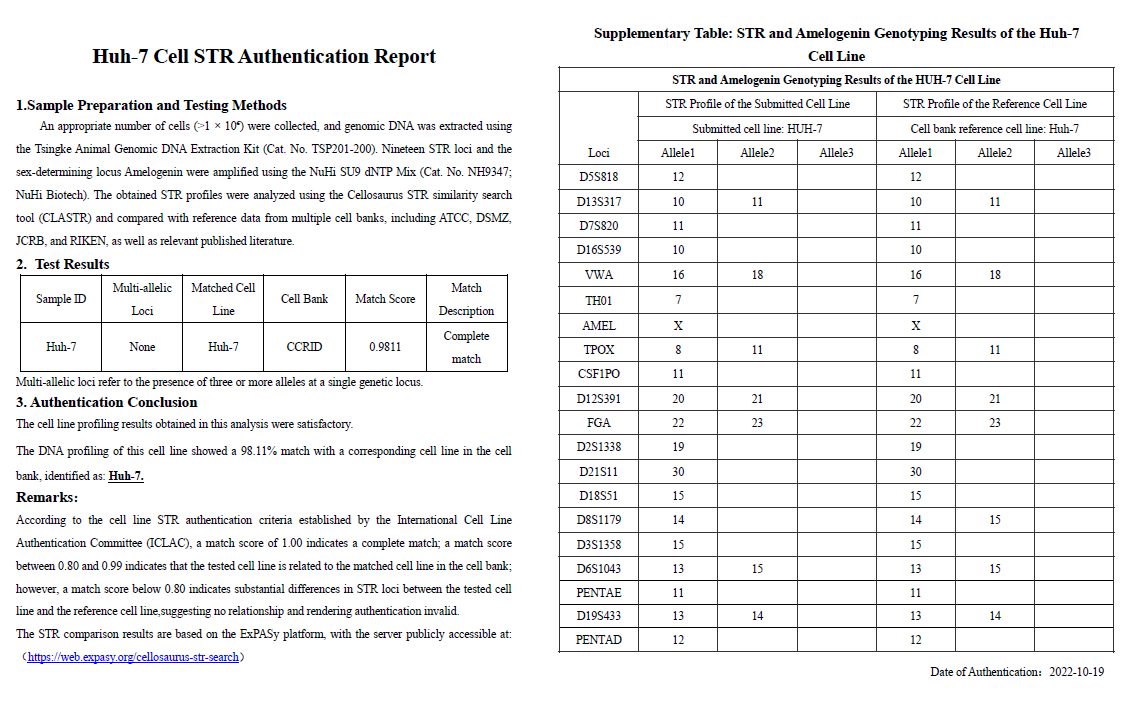

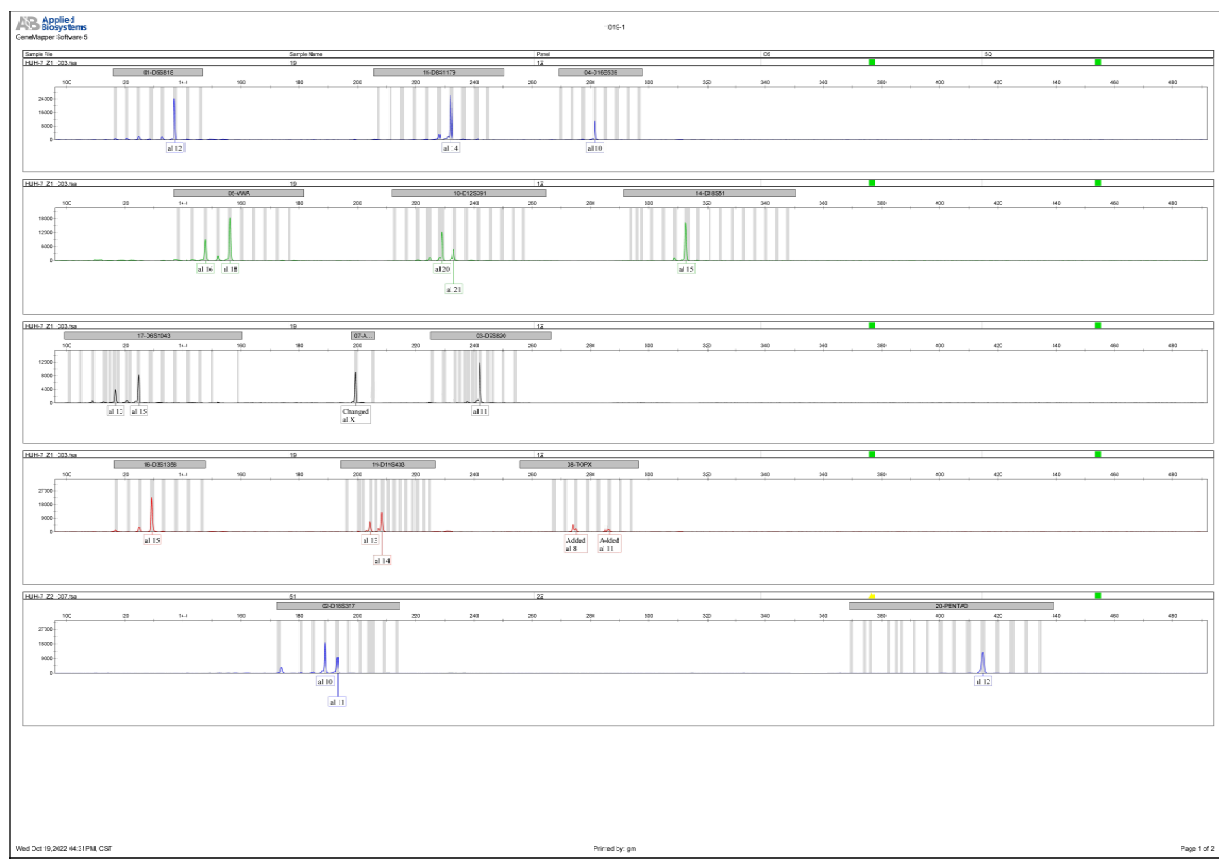

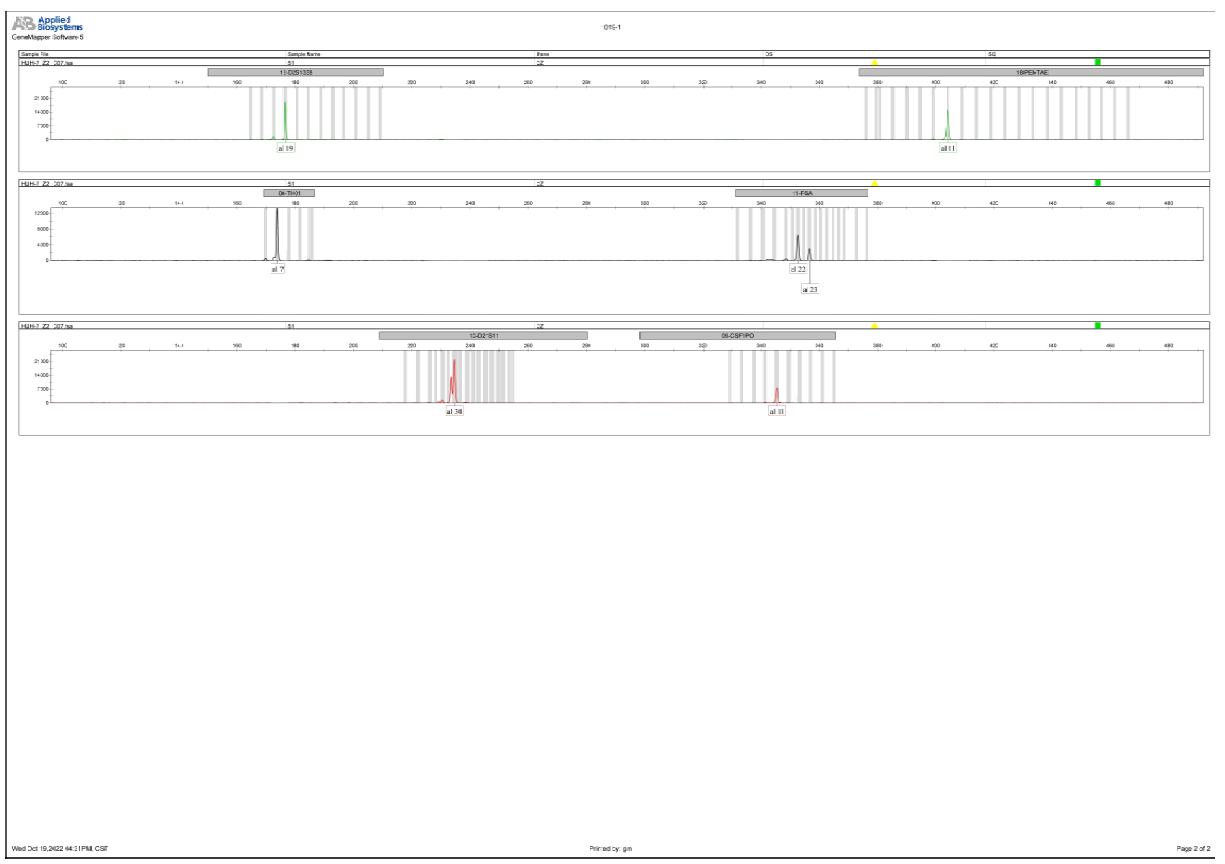


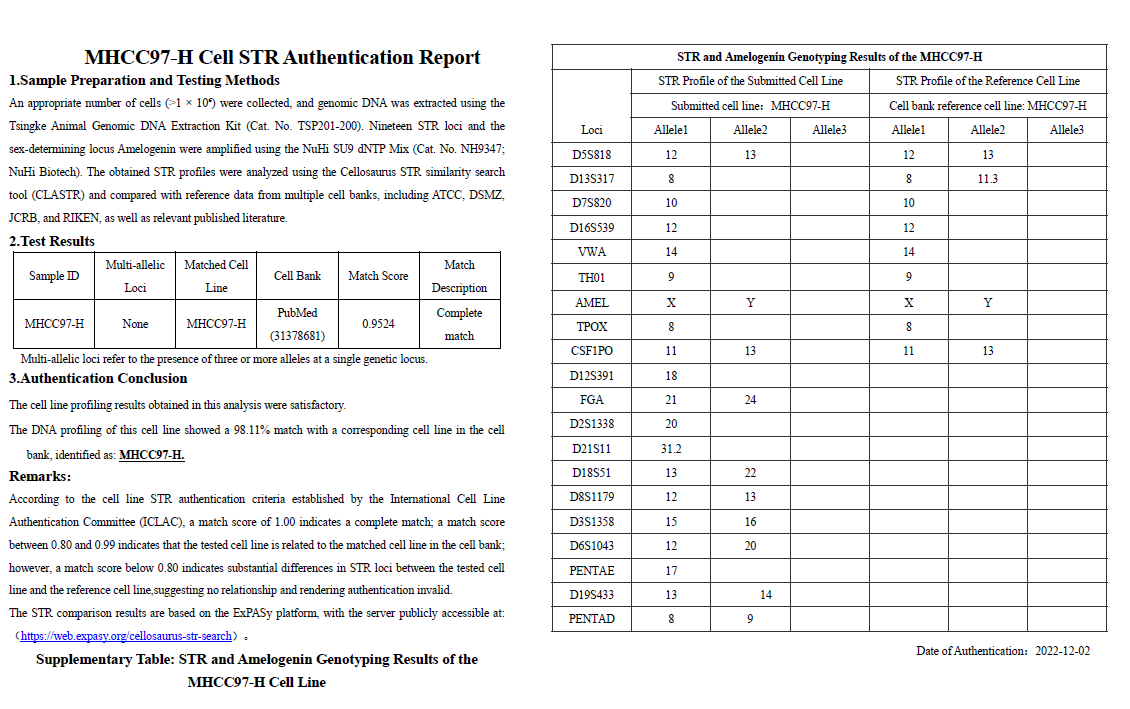

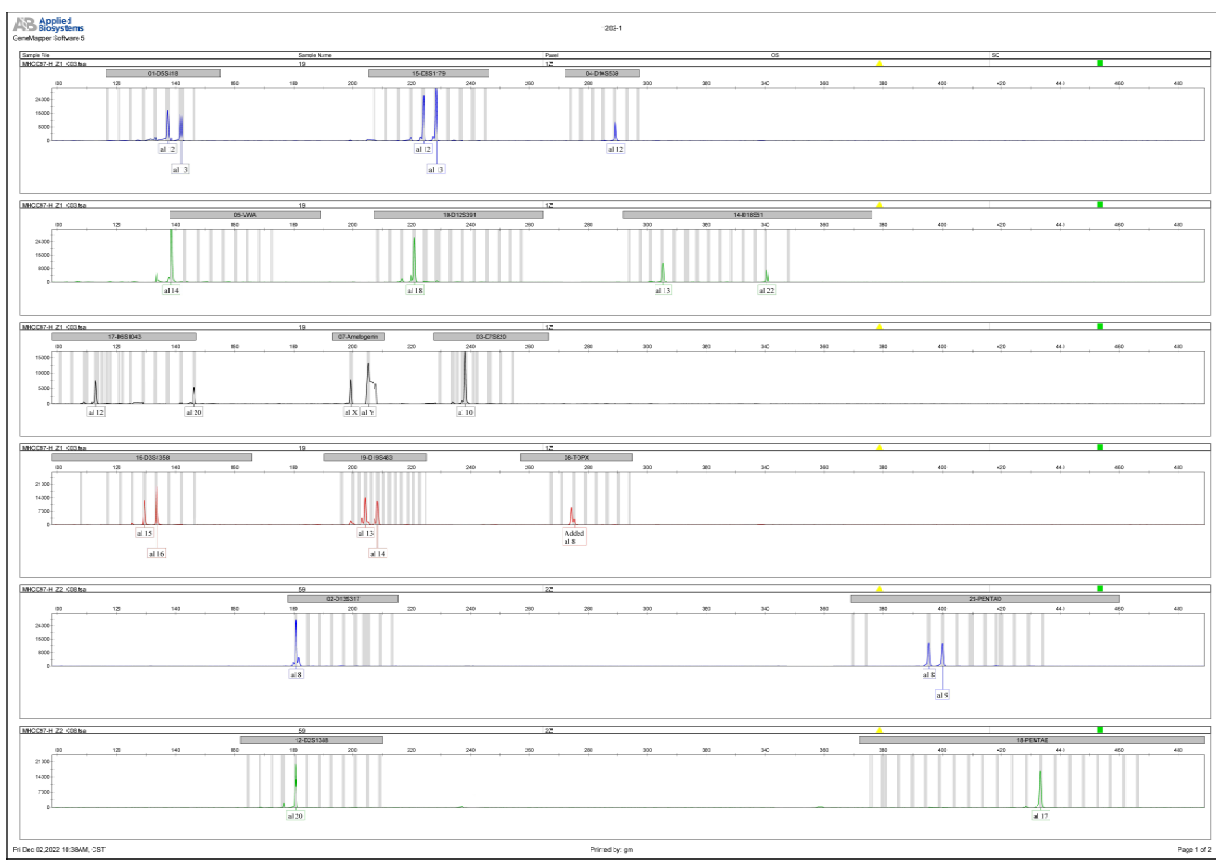

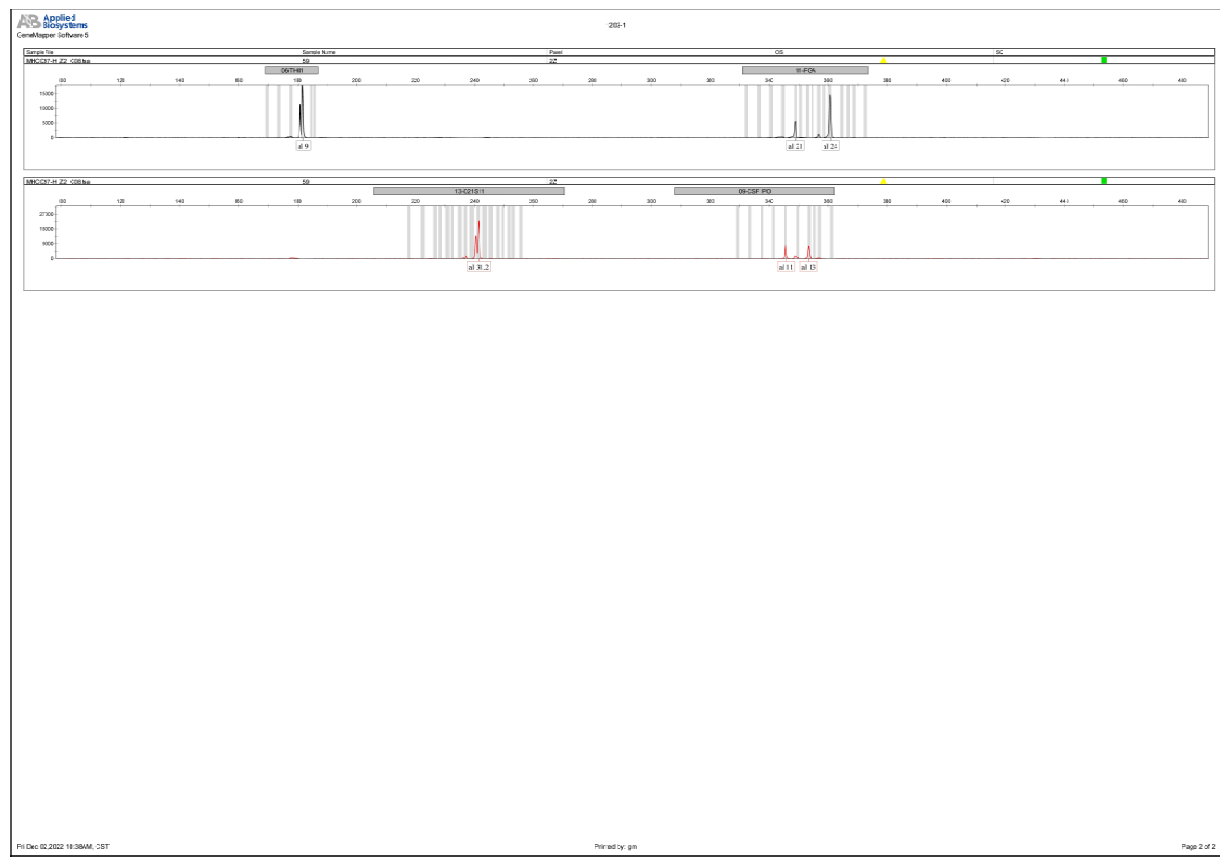

Supplement: Supplementary file 9 — STR [file 41419_2025_8393_MOESM9_ESM.docx]
