# Supplementary material for: CYP8B1 inhibits hepatocellular carcinoma progression by repressing PAK4 transcription through inhibition of nuclear translocation of u-STAT1
Source: Cell Death Dis. 2025 Dec 31;17(1):172. doi: 10.1038/s41419-025-08393-3 (PMC12876879; doi:10.1038/s41419-025-08393-3)
Supplement: Supplementary file 7 — Supplementary Material Legends [file 41419_2025_8393_MOESM7_ESM.docx]

Figure S1. (A) CCK-8 assay to evaluate the effects of CDCA and OCA at different concentrations on the proliferation of the hepatocellular carcinoma cell line Huh7. (B) The expression of FXR and TGR5. (C) CCK-8 assay validating sorafenib resistance in Huh7 and Huh7-SR cells, as reflected by IC_50_ values.

Table S1. Clinical characteristics of hepatocellular carcinoma (HCC) tissue microarray samples.

This table summarises the clinicopathological information of patients included in the HCC tissue microarray, including age, sex, tumour size, tumour grade, TNM stage and vascular invasion status.

Table S2. Primer sequences employed in this study.

This table lists all primer sequences utilised for quantitative real-time PCR and other molecular assays, including forward and reverse sequences.

Table S3. Predicted STAT1-binding motifs within the upstream regulatory region of the PAK4 gene.

This table lists the putative STAT1-binding sites identified within the genomic region upstream of the PAK4 transcription start site (NC_000019.10:39123786–39125785). The prediction results include the motif matrix ID, motif name, prediction score, relative score, genomic coordinates (start and end positions), DNA strand orientation, and the predicted binding sequence.

Table S4. Mass spectrometry–based identification of CYP8B1-associated proteins.

This table presents the mass spectrometry results obtained from CYP8B1 protein pull-down assays, including peptide counts, protein scores, sequence coverage, and identified interacting proteins.

Table S5. Predicted transcription factors regulating PAK4 expression across multiple databases.

This table integrates transcription factor predictions for PAK4 derived from multiple bioinformatics platforms.
